# Low Dose Histone Deacetylase Inhibitor, Depsipeptide (FR901228), Promotes Adenoviral Transduction in Human Rhabdomyosarcoma Cell Lines

**DOI:** 10.1080/13577140410001679220

**Published:** 2004-03

**Authors:** Fariba Navid, Blaine T. Mischen, Lee J. Helman

**Affiliations:** 1 Pediatric Oncology Branch National Cancer Institute National Institutes of Health Bethesda MD 20892-1928 USA; 2 Department of Hematology/Oncology St. Jude Children's Research Hospital 332 N. Lauderdale Street Memphis TN 38105-2974 USA

## Abstract

*Purpose*. Transduction of rhabdomyosarcoma (RMS) cells with adenoviral vectors for *in vivo* and *in vitro* applications has been limited by the low to absent levels of coxackie and adenovirus receptor (CAR). This study investigates the potential use of low doses of a histone deacetylase inhibitor, depsipeptide (FR901228), currently in Phase II human trials, to enhance adenoviral uptake in six rhabdomyosarcoma cell lines.

*Methods*. Differences in adenoviral uptake in the presence and absence of depsipeptide (FR901228) were assessed using an adenoviral construct tagged with green fluorescent protein. Changes in CAR and α_v_ integrin expression RMS in response to pretreatment with depsipeptide (FR901128) was determined using RT-PCR.

*Results*. Pretreatment of five of six RMS cell lines with 0.5 ng/ml of depsipeptide (FR901228) for 72 h resulted in increased viral uptake as assessed by green fluorescent protein expression. RT-PCR analysis for CAR showed that in four of these five cell lines, CAR expression was increased 2.8–8.1-fold in cells treated with depsipeptide (FR901228) as compared to control. α_v_ integrin expression was substantially increased in the one cell line, RH5, which showed increased GFP expression in response to depsipeptide (FR901228) pretreatment but a minimal increase in CAR expression.

*Conclusions*. Depsipeptide (FR901228) can be used as a vehicle to enhance adenoviral transduction in a majority of RMS cells. The mechanism of increased viral uptake appears to mediate via upregulation of CAR.


*Sarcoma*, March 2004, *VOL. 8, NO. 1*, 25–30

Taylor & Francis healthsciences

ORIGINAL ARTICLE

### Low dose histone deacetylase inhibitor, depsipeptide (FR901228), promotes adenoviral transduction in human rhabdomyosarcoma cell lines

FARIBA NAVID, BLAINE T. MISCHEN & LEE J. HELMAN

*Pediatric Oncology Branch, National Cancer Institute, National Institutes of Health, Bethesda, MD 20892-1928, USA*

**Abstract**

*Purpose.* Transduction of rhabdomyosarcoma (RMS) cells with adenoviral vectors for *in vivo* and *in vitro* applications has been limited by the low to absent levels of coxackie and adenovirus receptor (CAR). This study investigates the potential use of low doses of a histone deacetylase inhibitor, depsipeptide (FR901228), currently in Phase II human trials, to enhance adenoviral uptake in six rhabdomyosarcoma cell lines.

*Methods.* Differences in adenoviral uptake in the presence and absence of depsipeptide (FR901228) were assessed using an adenoviral construct tagged with green fluorescent protein. Changes in CAR and $\alpha_v$ integrin expression RMS in response to pretreatment with depsipeptide (FR901128) was determined using RT-PCR.

*Results.* Pretreatment of five of six RMS cell lines with 0.5 ng/ml of depsipeptide (FR901228) for 72 h resulted in increased viral uptake as assessed by green fluorescent protein expression. RT-PCR analysis for CAR showed that in four of these five cell lines, CAR expression was increased 2.8–8.1-fold in cells treated with depsipeptide (FR901228) as compared to control. $\alpha_v$ integrin expression was substantially increased in the one cell line, RH5, which showed increased GFP expression in response to depsipeptide (FR901228) pretreatment but a minimal increase in CAR expression.

*Conclusions.* Depsipeptide (FR901228) can be used as a vehicle to enhance adenoviral transduction in a majority of RMS cells. The mechanism of increased viral uptake appears to mediate via upregulation of CAR.

**Key words:** *histone deacetylase inhibitor, adenoviral uptake, rhabdomyosarcoma*

### Introduction

Rhabdomyosarcoma (RMS) is the most common soft tissue sarcoma in childhood. Novel treatments are required to improve the poor survival rates of patients that present with advanced disease. Gene therapy, using adenoviral constructs, is one potential therapeutic strategy. Adenoviral infections are advantageous because of their ability to infect both dividing and nondividing cells, their large packaging capacity and their ability to propagate high-titer viral stocks. However, the relatively low efficiency of recombinant gene transfer to tumor cells, including RMS cells, has been a disadvantage to the use of adenovirus in gene therapy.

Adenoviral infection of a cell requires the binding of the knob domain of the fiber capsid protein of the virus to the primary cellular receptor, coxackie and adenovirus receptor (CAR).[1] The attachment the virus to CAR may be facilitated by heparin sulfate proteoglycans or sialic acid.[2,3] Subsequent internalization of the virion by receptor-mediated endocytosis is potentiated by the interaction of RGD peptide sequences in the penton base of the virus with secondary host cell receptors, integrins $\alpha_v\beta_3$ and $\alpha_v\beta_5$.[4] In addition, adenoviral entry requires the activation of the phosphatidylinositol-3-OH kinase and the Rho family GTPase signaling pathways which target the actin cytoskeleton.[5] How actin facilitates internalization of the virus is unknown.

A number of studies have reported that cancer cells express only low levels of CAR and are therefore poorly infected by adenovirus.[6–8] Using flow cytometry and immunohistochemistry, Cripe *et al.* have shown that RMS cell lines and primary tumor samples, respectively, have low to absent levels of CAR but adequate $\alpha_v$ integrin levels. Also, increasing CAR levels through transfection of CAR cDNA into RMS cells rendered them highly susceptible to adenovirus infection.[9] In a number of cell types, methods to achieve CAR-independent gene transfer have been utilized. These include modifications in adenoviral vectors as well as the use of polycations to alter the electrostatic repulsion between the negatively

Correspondence to: Fariba Navid, M.D., Department of Hematology/Oncology, St. Jude Children's Research Hospital, 332 N. Lauderdale Street, Memphis, TN 38105-2974, USA. Tel.: + 1-901-495-2573; Fax: + 1-901-521-9005; E-mail: fariba.navid@stjude.org

1357-714X print/1369–1643 © 2004 Taylor & Francis Ltd
DOI: 10.1080/13577140410001679220

charged cell surface and adenoviral particles.[6,9–13] An alternative strategy would be to increase the level of CAR in tumor cells.

Depsipeptide (FR901228), is a histone deacetylase inhibitor isolated from *Chromobacterium violaceum* No. 968.[14,15] By increasing the acetylation of histones and thereby activating transcription, histone deacetylase inhibitors (HDIs) modulate the expression of genes involved in a number of cellular processes including inhibition of cell growth, promotion of differentiation and reversion of a transformed phenotype.[16,17] Previous reports have shown that inhibitors of histone deacetylase, trichostatin A and butyrate, can increase adenoviral transgene expression at the transcriptional level.[18–20] Recently, Kitazono *et al.* demonstrated an increase in CAR, $\alpha_v$ integrin and adenovirus-mediated gene expression in several carcinoma cell lines and hematopoetic cells pretreated with depsipeptide (FR901228).[8,21] In the present study, we describe the use of low dose depsipeptide to enhance adenoviral transduction in a panel of six rhabdomyosarcoma cell lines.

### Materials and Method

### *Cell culture and reagents*

Rh30, Rh5, Rh13 and Rh18 are cell lines cultured from human alveolar rhabdomyosarcoma tumors.[22] RD4a is a subclone of a well established human embryonal rhabdomyosarcoma cell line, RD.[23] CTR (obtained from Dr. Maria Tsokos, National Cancer Institute, Bethesda, MD) is a human embryonal rhabdomyosarcoma cell line.[24] All cell lines were cultured in RPMI 1640 with 10% fetal bovine serum (FBS), 100 U/ml of penicillin, 100 μg/ml streptomycin and 2 mM L-glutamine at 37°C and 5–6% $CO_2$.

Depsipeptide, FR901228, was obtained from the Pharmaceutical Management Branch, Cancer Therapy Evaluation Program, National Cancer Institute (Bethesda, MD).

### *MTT assay*

Cells were plated in triplicate in 96-well plates at a density of $5 \times 10^3$/well. Twenty-four hours later, media was removed and replaced with media containing various concentrations of depsipeptide (FR901228). Control cells were treated with identical concentrations of DMSO (diluent for depsipeptide). After 72 h, cell number was determined using colorimetric MTT (3-(4,5-dimethylthiazol-2-yl)-2,5-diphenyltetrazolium bromide) (Sigma Chemical Co., St. Louis, MO) assay as previously described.[25,26] Absorbencies were measured on a Titertek enzyme-linked immunosorbent assay reader using a test wavelength of 570 nm and a reference wavelength of 690 nm. The optical density measurements were converted to cell number using a standard curve.

### *Adenoviral infection*

RMS cells were plated at a density of $2.5 \times 10^5$ cells in six-well plates. After 24 h, 0.5 ng/ml of depsipeptide (FR901228) was added to designated wells and DMSO to the control wells. Seventy-two hours later the cells were incubated for 1 h with equal amounts of adenovirus, AdU1, in serum free media. AdU1 is an E1 and E3 deleted replication defective type 5 adenovirus with a GFP cassette driven by a CMV promoter.[27] AdU1 titre of $1 \times 10^{11}$ $TCID_{50}$/ml was determined by the $TCID_{50}$ assay as described by Qbiogene (Carlsbad, CA) except that the presence of GFP-positive cells instead of cytopathy was used as an indicator of viral infection.

### *Western blot analysis*

Cells were washed twice with 1 × PBS and then lysed with Lamelli buffer (0.125 M Tris, 20% glycerol, 4% SDS, pH 6.8) and protease inhibitors (Complete Mini, EDTA free, protease inhibitor cocktail tablets (Roche Diagnostic Corporation, Indianapolis, IN). Protein concentration was determined using Bio-Rad DC Protein Assay (Bio-Rad Laboratories, Hercules, CA). Ten μg of protein were resolved on a NOVEX 4–20% Tris–glycine gel (Invitrogen Corporation, Carlsbad, CA) and transferred onto a nitrocellulose membrane overnight at 20 V using a wet electroblotting system. Membranes were probed with rabbit anti-human GFP antibody protein (1:5000) (Invitrogen) and mouse monoclonal anti-human α-tubulin (0.1 μg/ml) (Ab-1) (Oncogene Research Products, Cambridge, MA). Nuclear protein was isolated to assess levels of histone H3 and acetylated histone H3. RMS cells treated for 72 h with depsipeptide or DMSO were washed twice with 1 × PBS. The cells were lysed in 10 mM Hepes (pH 7.9), 1.5 mM $MgCl_2$, 10 mM KCl, 0.5 mM DTT and protease inhibitors and spun at 12 000 rpm for 15 min at 4°C. Supernatant was removed and 50 μl of 50 mM Hepes, 420 mM KCl, 0.1 mM EDTA (pH 8), 5 mM $MgCl_2$, 0.5 mM DTT, and 20% glycerol were added and subjected to three cycles of freezing/thawing. The mixture was spun at 12 000 rpm for 15 min at 4°C. Equal volumes of supernatant and Lamelli buffer were loaded onto a 4–20% Tris–glycine gel and transferred as described above. Blots were probed with rabbit polyclonal anti-histone H3 (2 μg/ml) (catalog 06-755) (Upstate Biotechnology, Lake Placid, NY) and rabbit polyclonal anti-acetyl histone H3 (1 μg/ml) (catalog 06-911) (Upstate Biotechnology).

### *RT-PCR*

RT-PCR was performed using Ready-To-Go RT-PCR beads (Amersham Pharmacia Biotech, Inc., Piscataway, NJ). Each reaction was performed using

1 μg RNA, 0.5 μg of pd(T)$_{12-18}$ and 0.2 μM of the following gene specific primers and PCR conditions. Human CAR primers: sense 5′-GATCAGTGCC TGTTGCGTCTA and antisense 5′-TCACAGG AATCGCACCCA (product size = 399 bp); 42°C 30 min, 95°C 5 min, 30 cycles 95°C 30 s, 55°C 30 s, 72°C 1 min, 1 cycle 72°C 10 min. Human $\alpha_v$ integrin primers:[8] sense 5′-TAAAGGCACATGGCAAAGG AGT and antisense 5′-CAGTGGAAATGGAAA CGATGAGC (product size = 490 bp): 42°C 30 min, 95°C 5 min, 28 cycles 94°C 20 s, 64°C 40 s, 72°C 1 min, 1 cycle 72°C 10 min. Human GAPDH primers: sense 5′-AAGGTCGGAGTCAA CGGATT and antisense 5′-CATTGATGACAAGC TTCCCG. The PCR products were cloned and sequenced to verify that the amplified product was the gene of interest.

### Results and Discussion

We first determined the effect of various concentrations of depsipeptide (FR901228) on six RMS cell lines since depsipeptide (FR901228) has been shown to have antitumor activity as well as alter the growth of tumor cells. The growth inhibition of RMS cells treated with ≤ 0.5 ng/ml depsipeptide (FR901228) for 72 h was less than 20% compared to controls (Fig. 1a). Since our purpose was to use depsipeptide to modulate the efficiency of viral infection in these tumor cells and not to use the drug for its antitumor activity, we conducted the subsequent experiments with 0.5 ng/ml of depsipeptide (FR901228). As shown in Fig. 1b for the CTR cell line, an increase in acetylated histone H3 occurs at this low dose of depsipeptide (FR901228).

In order to study the ability of depsipeptide (FR901228) to enhance adenovirus-mediated gene transfer in RMS cells, we infected six RMS cell lines with an adenoviral vector containing a CMV promoter driven green fluorescent protein (GFP) gene. Cells infected with this virus can be visualized under a fluorescent microscope and GFP can be quantitated by Western analysis. As shown in Fig. 2a, five of the six cell lines pretreated with depsipeptide (FR901228) had increased GFP expression compared to the untreated control cells. The degree of GFP protein expression, quantitated by Western blot analysis, was 8.6–2.6-fold greater in

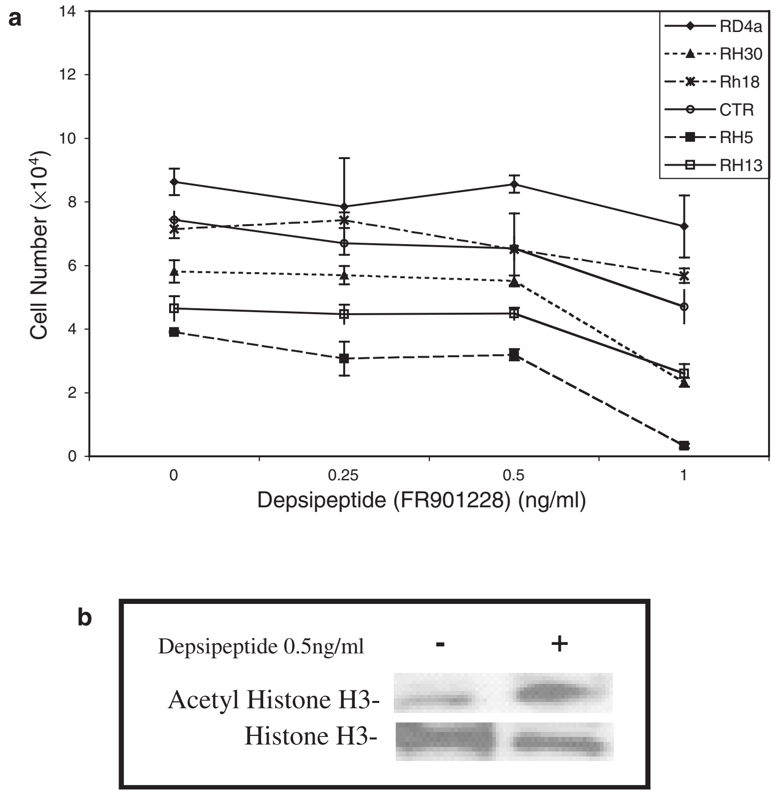

**Fig. 1.** *Effect of depsipeptide (FR901228) on the growth of RMS cell lines and acetylation of histone H3. (a) Six RMS cell lines (RD4a, CTR, RH5, RH13, RH18, and RH30) were plated in triplicate in a 96-well plate at a density of 5 × 10$^3$cells/well. Twenty-four hours later, the cells were incubated with different concentrations of depsipeptide (FR901228) or DMSO (diluent for depsipeptide). Cells were harvested after 72 h of treatment. Cell number was determined using a colorimetric MTT assay. The bars indicated represent standard deviation from the mean of triplicate samples. (b) Western blot analysis for histone H3 acetylation in CTR cells in response to 72 h treatment with depsipeptide (+) or DMSO (−). Histone H3 serves as a control for loading of nuclear protein lysates.*

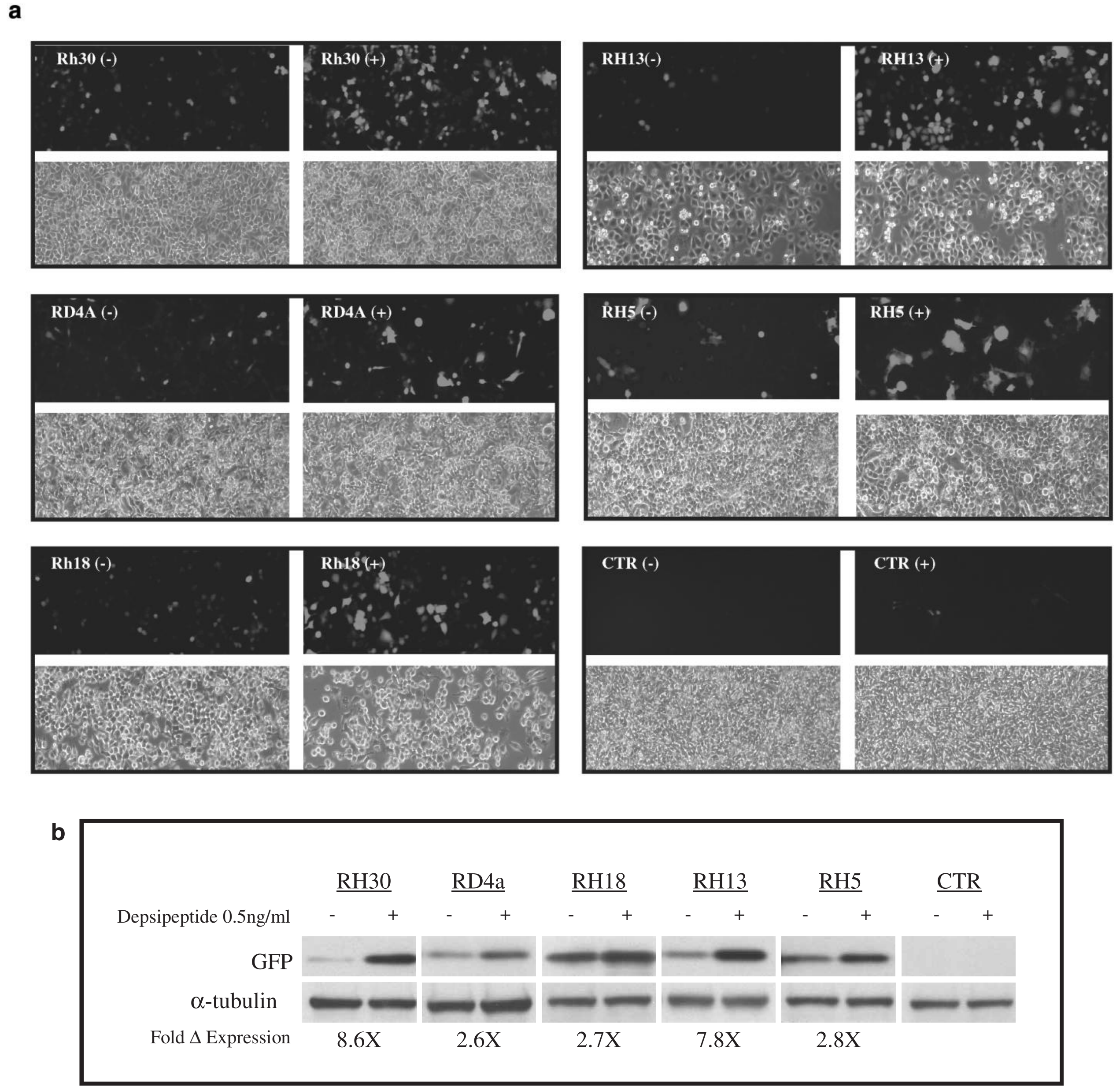

**Fig. 2.** *Adenoviral GFP expression in RMS cell lines pretreated with depsipeptide (FR901228). RMS cells were plated at a density of* $2.5 \times 10^5$ *cells in six-well plates. After 24 h, 0.5 ng/ml of depsipeptide (FR901228) was added to designated wells and DMSO to the control wells. Seventy-two hours later the cells were incubated for 1 h with equal amounts of adenovirus, AdU1, in serum free media. The cells were replenished with complete media. For each cell line pretreated with depsipeptide (FR901228) (+) or DMSO (–), fluorescent (top) and phase contrast (bottom) photographs were taken 72 h after adenoviral infection (a). Media was then removed and the cells were harvested for protein. Levels of green fluorescent protein expression were assessed using Western blot analysis. Densitometry was performed using NIH Image 1.60 software and was used to calculate the fold change in protein expression. α-Tubulin was used to normalize the protein loading.*

the depsipeptide-treated cells then in the untreated control cells (Fig. 2b). No GFP protein expression was detected in the CTR cell line.

Adenoviral infection is mainly dependent on the presence of CAR on the surface of normal as well as malignant cells. Because CAR expression has been reported to be low or sometimes absent in RMS cell lines and tissues, we investigated whether the increased adenoviral expression observed in the RMS cell lines treated with depsipeptide (FR901228) was due to increased CAR expression in these cell lines. Using RT-PCR, we were able to detect transcripts for CAR in five of six cell lines. In addition, four of six RMS cells treated with depsipeptide for 72 h showed a 2.8–8.1-fold increase in CAR expression compared to untreated cells (Fig. 3a). RH30 had the greatest increase in CAR expression in response to depsipeptide

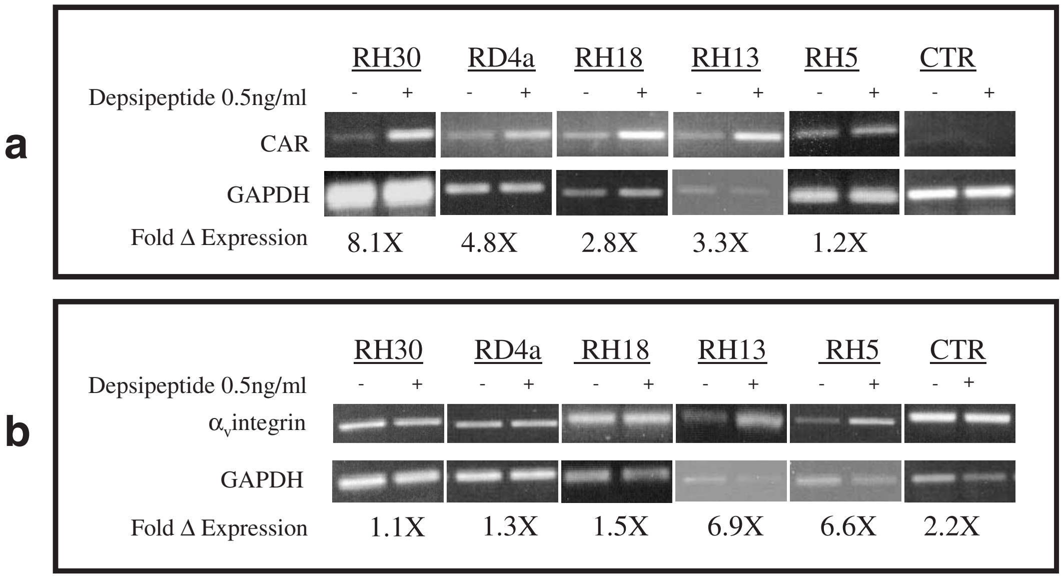

**Fig. 3.** *Effect of depsipeptide (FR901228) on RNA expression of CAR (a) and $\alpha_v$ integrin (b). RMS cells were treated for 72 h with 0.5 ng/ml of depsipeptide (FR901228) (+) or DMSO (−) and then harvested for RNA extraction. Levels of gene expression for human CAR, $\alpha_v$ integrin, and GAPDH were assessed using semi-quantitative RT-PCR. GAPDH was used to normalize the loading. Densitometry was performed using NIH Image 1.60 software and was used to calculate the fold change in protein expression.*

(FR901228), and showed the greatest increase in adenoviral uptake in response to depsipeptide (FR901228) pretreatment. Unlike Rh30, RH5 had minimally upregulated CAR expression in the presence of depsipeptide (FR901228); however, a 2.8-fold increase in adenoviral uptake in response to pretreatment with depsipeptide was observed. In contrast, $\alpha_v$ integrin expression was present in all six cell lines, and was modulated by depsipeptide (FR901228) 1.5–6.9-fold in four of the six RMS cell lines, including RH5 (Fig. 3b). The increased adenoviral uptake in RH5 cells in response to depsipeptide (FR901228) may be due to a 6.6-fold increase in $\alpha_v$ integrin. Even though we detected a 2-fold increase $\alpha_v$ integrin expression in the CTR cell line, we observed no viral uptake in this cell line. This is probably due to the lack of CAR expression in both the presence and absence of depsipeptide (FR901228), suggesting that the presence of CAR is critical for adenoviral gene transfer.

We conclude that depsipeptide (FR901228) modulates adenovirus transgene expression through upregulation of the viral receptor, CAR and in some cases $\alpha_v$ integrin, in RMS cells. The change in gene expression is variable between the different RMS cell lines. These findings are similar to what has been reported for carcinoma cell lines and hematopoetic cells.[8,21] The mechanism of increased gene expression is presumed to be release of transcription repression through inhibition of histone deacetylases. As an efficient means of exogenous gene expression, the addition of low dose depsipeptide (FR901228) to promote adenoviral uptake may be a simple and useful approach for *in vitro* studies of rhabdomyosarcoma cells. Since depsipeptide (FR901228) is currently in adult Phase II trials and in a pediatric Phase I trial, the use of this agent *in vivo* is plausible and could be used for the purpose of increasing the efficacy of adenoviral mediated gene therapy.

### Acknowledgements

The authors would like to thank Merrill Goldsmith and Tito Fojo for their helpful discussions.

### References

1. Bergelson JM, Cunningham JA, Droguett G, Kurt-Jones EA, Krithivas A, Hong JS, Horwitz MS, Crowell RL, Finberg RW. Isolation of a common receptor for Coxsackie B viruses and adenoviruses 2 and 5. *Science* 1997; 275: 1320–3.
2. Arnberg N, Edlund K, Kidd AH, Wadell G. Adenovirus type 37 uses sialic acid as a cellular receptor. *J Virol* 2000; 74: 42–8.
3. Dechecchi MC, Tamanini A, Bonizzato A, Cabrini G. Heparan sulfate glycosaminoglycans are involved in adenovirus type 5 and 2-host cell interactions. *Virology* 2000; 268: 382–90.
4. Wickham TJ, Mathias P, Cheresh DA, Nemerow GR. Integrins alpha v beta 3 and alpha v beta 5 promote adenovirus internalization but not virus attachment. *Cell* 1993; 73: 309–19.
5. Li E, Stupack DG, Brown SL, Klemke R, Schlaepfer DD, Nemerow GR. Association of p130CAS with phosphatidylinositol-3-OH kinase mediates adenovirus cell entry. *J Biol Chem* 2000; 275: 14729–35.
6. Miller CR, Buchsbaum DJ, Reynolds PN, Douglas JT, Gillespie GY, Mayo MS, Raben D, Curiel DT. Differential susceptibility of primary and established human glioma cells to adenovirus infection: targeting via the epidermal growth factor receptor achieves fiber receptor-independent gene transfer. *Cancer Res* 1998; 58: 5738–48.
7. Li Y, Pong RC, Bergelson JM, Hall MC, Sagalowsky AI, Tseng CP, Wang Z, Hsieh JT. Loss of adenoviral receptor expression in human bladder cancer cells: a potential impact on the efficacy of gene therapy. *Cancer Res* 1999; 59: 325–30.
8. Kitazono M, Goldsmith ME, Aikou T, Bates S, Fojo T. Enhanced adenovirus transgene expression in malignant cells treated with the histone deacetylase inhibitor FR901228. *Cancer Res* 2001; 61: 6328–30.
9. Cripe TP, Dunphy EJ, Holub AD, Saini A, Vasi NH, Mahller YY, Collins MH, Snyder JD, Krasnykh V, Curiel DT, Wickham TJ, DeGregori J, Bergelson JM, Currier MA. Fiber knob modifications overcome low, heterogeneous expression of the coxsackievirus-adenovirus receptor that limits adenovirus gene transfer and oncolysis for human rhabdomyosarcoma cells. *Cancer Res* 2001; 61: 2953–60.
10. Fasbender A, Zabner J, Chillon M, Moninger TO, Puga AP, Davidson BL, Welsh MJ. Complexes of adenovirus with polycationic polymers and cationic lipids increase the efficiency of gene transfer in vitro and in vivo. *J Biol Chem* 1997; 272: 6479–89.
11. Kaplan JM, Pennington SE, St George JA, Woodworth LA, Fasbender A, Marshall J, Cheng SH, Wadsworth SC, Gregory RJ, Smith AE. Potentiation of gene transfer to the mouse lung by complexes of adenovirus vector and polycations improves therapeutic potential. *Hum Gene Ther* 1998; 9: 1469–79.
12. Lanuti M, Kouri CE, Force S, Chang M, Amin K, Xu K, Blair I, Kaiser L, Albelda S. Use of protamine to augment adenovirus-mediated cancer gene therapy. *Gene Ther* 1999; 6: 1600–10.
13. Dunphy EJ, Redman RA, Herweijer H, Cripe TP. Reciprocal enhancement of gene transfer by combinatorial adenovirus transduction and plasmid DNA transfection in vitro and in vivo. *Hum Gene Ther* 1999; 10: 2407–17.
14. Nakajima H, Kim YB, Terano H, Yoshida M, Horinouchi S. FR901228, a potent antitumor antibiotic, is a novel histone deacetylase inhibitor. *Exp Cell Res* 1998; 241: 126–33.
15. Ueda H, Nakajima H, Hori Y, Fujita T, Nishimura M, Goto T, Okuhara M. FR901228, a novel antitumor bicyclic depsipeptide produced by Chromobacterium violaceum No. 968. I. Taxonomy, fermentation, isolation, physico-chemical and biological properties, and antitumor activity. *J Antibiot (Tokyo)* 1994; 47: 301–10.
16. Ueda H, Nakajima H, Hori Y, Goto T, Okuhara M. Action of FR901228, a novel antitumor bicyclic depsipeptide produced by Chromobacterium violaceum no. 968, on Ha-ras transformed NIH3T3 cells. *Biosci Biotechnol Biochem* 1994; 58: 1579–83.
17. Ueda H, Manda T, Matsumoto S, Mukumoto S, Nishigaki F, Kawamura I, Shimomura K. FR901228, a novel antitumor bicyclic depsipeptide produced by *Chromobacterium violaceum* No. 968. III. Antitumor activities on experimental tumors in mice. *J Antibiot (Tokyo)* 1994; 47: 315–23.
18. Dion LD, Goldsmith KT, Tang DC, Engler JA, Yoshida M, Garver RI Jr. Amplification of recombinant adenoviral transgene products occurs by inhibition of histone deacetylase. *Virology* 1997; 231: 201–9.
19. Tang DC, Johnston SA, Carbone DP. Butyrate-inducible and tumor-restricted gene expression by adenovirus vectors. *Cancer Gene Ther* 1994; 1: 15–20.
20. Gaetano C, Catalano A, Palumbo R, Illi B, Orlando G, Ventoruzzo G, Serino F, Capogrossi MC. Transcriptionally active drugs improve adenovirus vector performance in vitro and in vivo. *Gene Ther* 2000; 7: 1624–30.
21. Kitazono M, Rao VK, Robey R, Aikou T, Bates S, Fojo T, Goldsmith ME. Histone deacetylase inhibitor FR901228 enhances adenovirus infection of hematopoietic cells. *Blood* 2002; 99: 2248–51.
22. Houghton JA, Houghton PJ, Webber BL. Growth and characterization of childhood rhabdomyosarcomas as xenografts. *J Natl Cancer Inst* 1982; 68: 437–43.
23. Kalebic T, Judde JG, Velez-Yanguas M, Knutsen T, Helman LJ. Metastatic human rhabdomyosarcoma: molecular, cellular and cytogenetic analysis of a novel cellular model. *Invasion Metastasis* 1996; 16: 83–96.
24. Felix CA, Kappel CC, Mitsudomi T, Nau MM, Tsokos M, Crouch GD, Nisen PD, Winick NJ, Helman LJ. Frequency and diversity of p53 mutations in childhood rhabdomyosarcoma. *Cancer Res* 1992; 52: 2243–7.
25. Denizot F, Lang R. Rapid colorimetric assay for cell growth and survival. Modifications to the tetrazolium dye procedure giving improved sensitivity and reliability. *J Immunol Methods* 1986; 89: 271–7.
26. El-Badry OM, Romanus JA, Helman LJ, Cooper MJ, Rechler MM, Israel MA. Autonomous growth of a human neuroblastoma cell line is mediated by insulin-like growth factor II. *J Clin Invest* 1989; 84: 829–39.
27. Abounader R, Lal B, Luddy C, Koe G, Davidson B, Rosen EM, Laterra J. In vivo targeting of SF/HGF and c-met expression via U1snRNA/ribozymes inhibits glioma growth and angiogenesis and promotes apoptosis. *FASEB J* 2002; 16: 108–10.
